# Musculoskeletal Geometry, Muscle Architecture and Functional Specialisations of the Mouse Hindlimb

**DOI:** 10.1371/journal.pone.0147669

**Published:** 2016-04-26

**Authors:** James P. Charles, Ornella Cappellari, Andrew J. Spence, John R. Hutchinson, Dominic J. Wells

**Affiliations:** 1 Neuromuscular Diseases Group, Comparative Biomedical Sciences, Royal Veterinary College, 4 Royal College Street, London, NW1 0TU, United Kingdom; 2 Structure and Motion Lab, Comparative Biomedical Sciences, Royal Veterinary College, Hawkshead Lane, Hatfield, Hertfordshire, AL9 7TA, United Kingdom; 3 Department of Bioengineering, College of Engineering, Temple University, 1947 N. 12th Street, Philadelphia, PA 19122, United States of America; University of Sydney, AUSTRALIA

## Abstract

Mice are one of the most commonly used laboratory animals, with an extensive array of disease models in existence, including for many neuromuscular diseases. The hindlimb is of particular interest due to several close muscle analogues/homologues to humans and other species. A detailed anatomical study describing the adult morphology is lacking, however. This study describes in detail the musculoskeletal geometry and skeletal muscle architecture of the mouse hindlimb and pelvis, determining the extent to which the muscles are adapted for their function, as inferred from their architecture. Using I_2_KI enhanced microCT scanning and digital segmentation, it was possible to identify 39 distinct muscles of the hindlimb and pelvis belonging to nine functional groups. The architecture of each of these muscles was determined through microdissections, revealing strong architectural specialisations between the functional groups. The hip extensors and hip adductors showed significantly stronger adaptations towards high contraction velocities and joint control relative to the distal functional groups, which exhibited larger physiological cross sectional areas and longer tendons, adaptations for high force output and elastic energy savings. These results suggest that a proximo-distal gradient in muscle architecture exists in the mouse hindlimb. Such a gradient has been purported to function in aiding locomotor stability and efficiency. The data presented here will be especially valuable to any research with a focus on the architecture or gross anatomy of the mouse hindlimb and pelvis musculature, but also of use to anyone interested in the functional significance of muscle design in relation to quadrupedal locomotion.

## Introduction

Despite the widespread use of mice (*Mus musculus*) in studies investigating pre-clinical drug treatments and/or locomotor behaviour [1–5], the soft tissue anatomy of their musculoskeletal system has yet to be fully described. The mouse hindlimb, specifically, is a common area of interest for several reasons. First, the muscles are known to show obvious histopathology in models of various neuromuscular diseases [6–8], and as such are good subjects in which to study treatment efficacy. Second, many of the muscles are close analogues to those of the human lower limb, so this work could provide valuable information and insight into the control of the musculoskeletal system in humans. Finally, the hindlimbs of mice are reasonably close to the inferred ancestral morphology (and body size) of placental or other mammals, and thus might serve as useful homologues/analogues for the evolution of mammalian locomotion [9]. Therefore, improving knowledge of the internal structure of these hindlimb muscles, as well as their functional roles in movement, will support both basic science and medicine. Previous detailed studies into the skeletal muscle anatomy of the mouse hindlimb have generally focused more on the embryology and development of the muscles [10] or comparing the relative effects of architecture and fibre types on determining contractile properties [11], rather than their geometry or functional specialisations. The architecture of the mouse forelimb muscles was described recently by Mathewson et al. [12], who compared the force-generating properties of the mouse muscles to homologues in the human arm. The musculoskeletal geometry and functional specialisations of these muscles were not investigated, however. As yet, there has not been a comprehensive treatment of the hindlimb anatomy of adult mice, complete with muscle architecture data, musculoskeletal geometry and assessments of the functional specialisations in the limb.

Skeletal muscle architecture refers to the arrangement of fibres within a muscle relative to its force-generating axis [13], and is known to have a major impact on force-generating properties, and by extension, function [11, 12, 14]. Important architectural variables include fibre length (L_f_), physiological cross-sectional area (PCSA) and fibre pennation angle (θ). Fibre length is a representation of the sum of in-series sarcomeres within a fibre, and has been shown to be directly proportional to muscle excursion (i.e. the distance a muscle shortens when contracted) and velocity of contraction [15]. PCSA represents the sum of all the fibres’ cross-sectional areas within a muscle, and is proportional to maximum force output [16]. Fibre pennation refers to the angle between a muscle’s fibres and its internal tendon or main line of action. A pennate fibre arrangement increases the number of muscle fibres which attach to this internal tendon for a given cross-sectional area, functioning to increase the number of fibres within a muscle, and therefore its PCSA. However, any increase in force this may provide is negated by the fact that these fibres are orientated at an angle to the muscle’s line of action, which means that muscles with these fibre arrangements produce less force relative to a muscle with no fibre pennation but the same mass and fibre length. Therefore, despite its perception as an important architectural characteristic of a muscle, it is unlikely that pennation angle itself has much effect on absolute muscle force production. It is possible, however, than fibre pennation allows muscles to uniquely distribute their mass relative to parallel fibred muscles, with their muscle bellies located more proximally, which taper distally to connect to external tendons [13, 17].

Several studies have noted that within certain functional groups, muscles have evolved to become specifically suited to their functions through their architectural design. For example, studies in cats [18], guinea pigs [16] and rabbits [19] have shown that the hamstring muscles, a bi-articular group of muscles which act around both the hip and the knee, have significantly longer absolute and relative fibre lengths and smaller PCSA values compared to the quadriceps, a powerful muscle group which performs an antagonistic function at the knee joint. These findings illustrate how different functional designs can exist within two muscle groups that act around the same joint. These differing functional adaptations, along with neural activity of sensory and motor neurons, allow motor control to be finely tuned during locomotion. For example, some muscles, such as the gastrocnemius group (ankle extensors), provide forceful, often quasi-isometric contractions while storing elastic energy in compliant tendons [20, 21], whereas others, such as the hamstrings (hip extensors and knee flexors), function to produce fast contractions and joint control [22]. The extent to which these specialisations exist within the functional groups of the mouse hindlimb (here including the pelvis) has yet to be elucidated. With this knowledge, testable hypotheses could be made for muscle function, and in the long term provide a strong link between detailed musculoskeletal structure and behaviour.

However, a muscle’s function is not only governed by the arrangement of its fibres. Musculoskeletal geometry, which refers to a muscle’s attachment points onto the skeleton and its respective path of action, also greatly affects its force-generating potential. Geometry affects muscular moment arms, which determine the effectiveness in which muscles generate force and produce joint rotations. Therefore when considering the functional anatomy of a musculoskeletal system, it is important to determine sites of muscle attachment and the directions in which these muscles act to the greatest degree of accuracy possible. We have recently made such representations in a three-dimensional (3D) musculoskeletal model of the mouse hindlimb and pelvis [17].

Due to the small size of the mouse hindlimb and the fragility of its muscles, simple manual dissection was found inadequate for determining attachment points. Therefore, we applied the recently developed technique of contrast-enhanced microCT scanning to provide a non-destructive alternative for discerning delicate soft tissue anatomy. This potentially reduces the risk of damaging important structures and allows specimens to be analysed in a digital context [23, 24]. Standard microCT scanning is a long established technique, however due to the low x-ray attenuation of soft tissue structures, discerning the internal organisation of skeletal muscle or internal organs is often not possible. Staining soft tissue samples in an aqueous solution of iodine potassium iodide (I_2_KI) prior to scanning eliminates these drawbacks [23–31]. This technique has since been used to visualise the skull musculature of rodents [26, 27], the Common Buzzard [30] and the American Alligator (*Alligator mississippiensis*) [29], as well as the penises of bats [25, 31] and the syringeal muscles of birds [32], among other applications. It produces images analogous to those from magnetic resonance imaging (MRI).

I_2_KI enhanced microCT scanning was used here to allow the bones and musculature of the mouse hindlimb to be clearly observed, subsequently digitally segmented and finally assembled into an interactive 3D model. With this model, it was possible for the musculoskeletal geometry of the mouse hindlimb and pelvis to be discerned and viewed non-destructively in a digital framework.

Our aim in this paper was to provide a detailed anatomical quantification of the musculoskeletal geometry and muscle architecture of the hindlimb and pelvis in mice. First, contrast-enhanced microCT scanning was used to identify muscles of the hindlimb and elucidate their geometry. Once identified, the internal architecture of these muscles was determined, and statistical analyses were used to investigate the extent to which the functional groups of the mouse hindlimb are uniquely specialised for one of their primary functional roles, locomotion.

## Materials and Methods

### Musculoskeletal Geometry

#### MicroCT scanning

**Fig 1:**
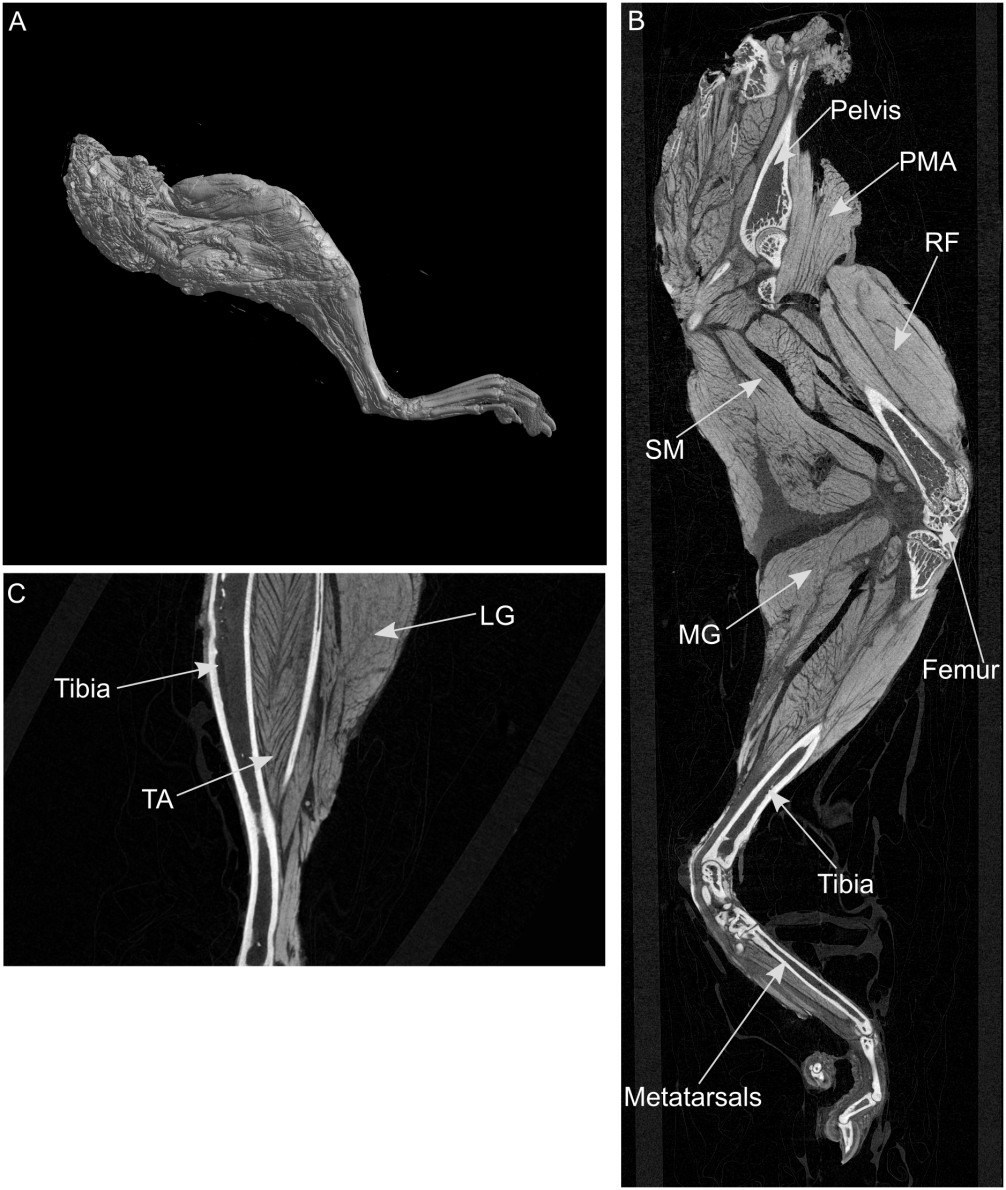
Reconstructed images of a mouse hindlimb following I_2_KI enhanced microCT scanning. A, medial view; B, mid-sagittal section of the whole limb; C, mid-sagittal section of distal leg. For muscle abbreviations, see Tables 1 and 2.

In preparation for I_2_KI enhanced microCT scanning, one C57BL/6 mouse (female, body mass 24.9g, age 117 days) was euthanized by cervical dislocation, and its right hindlimb was removed, skinned and immediately frozen. The limb was then thawed and placed in 10% neutral-buffered formalin (NBF) (HT501128-4L, Sigma) for 24hrs at room temperature to fix the soft tissue, and subsequently placed in 1x phosphate buffered saline (PBS) to remove residual fixative. The specimen was then immersed in an aqueous solution of 15% iodine-potassium iodide (I_2_KI, Lugols solution, Sigma, L6146) for eight days to enhance soft tissue contrast, and then placed in 70% ethanol solution until microCT scanning. Scanning was carried out using a SkyScan 1172 system (Bruker microCT, Belgium) at the following settings: 12.96μm resolution, 70kV, 141μA, Al 0.5mm filter. The images were reconstructed using NRecon software (Bruker microCT, Belgium), whereas images of the internal structures of the hindlimb were captured using CTvox software (Bruker microCT, Belgium) (Fig 1).

#### Segmentation

**Fig 2:**
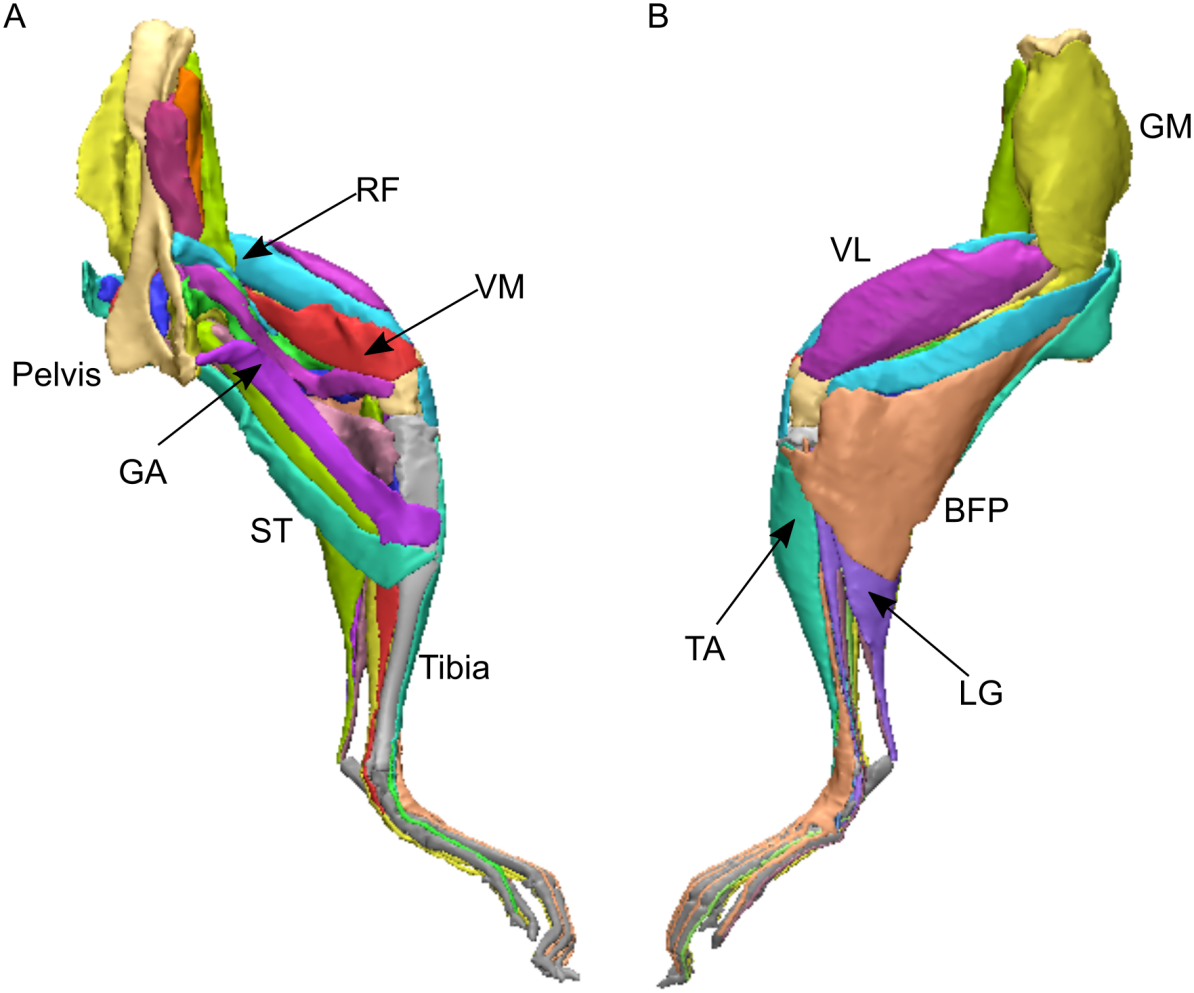
Three- dimensional representation of the mouse hindlimb, created through digital segmentation. A, medial view; B, lateral view. For muscle abbreviation, see Tables 1 and 2. This model can be viewed as an interactive 3D PDF (S1 Fig).

The muscles and bones of the mouse hindlimb were subsequently digitally segmented and rendered into discrete 3D meshes in Mimics software (Materialise Inc., Leuven, Belgium) using the reconstructed microCT images. These meshes were added together to create a 3D representation of the mouse hindlimb (Fig 2), which can be viewed as a 3D PDF (S1 Fig).

### Muscle architecture

#### Muscle collection

In order to gather architecture data for the muscles identified through digital segmentation, four adult C57BL/6 mice (female, body mass 23.45±2.73g, age 107.8±34.86 days; means ± S.D.) were euthanized by cervical dislocation, and both hindlimbs were removed at the pubic symphysis and skinned. The eight limbs were then placed in 10% NBF for 24 hours at room temperature to fix the soft tissue with the hip, knee, and ankle joints held at 90 degrees to maximise the potential of achieving optimal muscle fibre lengths throughout fixation. The limbs were then washed in 1x PBS to remove residual fixative, and stored in 1x PBS until dissection. Each muscle previously identified with digital segmentation was carefully dissected under high magnification to maximise the potential of removing the whole muscle at origin and insertion, and individually stored in 1x PBS until further measurement.

#### Measuring architecture

Six architectural variables were measured for each musculotendon unit (MTU): muscle (belly) mass (M_m_, mg), tendon mass (M_t_, mg), muscle (belly) length (L_m_, mm), tendon length (L_t_, mm), optimal fibre length (L_f_, mm) and fibre pennation angle (θ; °).

To determine muscle mass, each muscle was removed from 1x PBS and gently blotted dry, then placed on a fine electronic balance accurate to 0.01mg (Salter). Tendon mass (if applicable) was also determined with an identical technique. The measurement of both these variables was repeated three times for each muscle, and a mean value as well as standard deviations calculated. Muscle (belly) length was defined as the distance from the origin of the most proximal muscle fibres to the insertion of the most distal fibres. MTU length was defined as the distance from proximal tendon or muscle fibre origin to the insertion of the tendon or most distal fibres, and the tendon length of tendinous muscles was that MTU length minus the muscle (belly) length. Pennation angle was measured as the angle of muscle fibres relative to the internal tendon or aponeurosis. Muscle and tendon lengths as well as pennation angles for each individual MTU were measured with ImageJ software (U.S. National Institutes of Health, Bethesda, MD, USA), accurate to 0.01mm and 0.01° respectively. The length measurements were repeated three times for each muscle, with mean values as well as standard deviations calculated. Pennation angles were measured at five different areas of each muscle, with the mean of these values assumed to represent the average pennation angle of the muscle fibres. The standard deviations of these values were also calculated.

To determine muscle fibre length, muscles were placed in 2M sulphuric acid (H_2_SO_4_) solution (10558620, Fisher Scientific) for 24hrs to digest the surrounding connective tissue to allow for the extraction of fibre bundles or fascicles, consisting of ~5–10 fibres, of which five were removed for each muscle. Despite the possibility that some fibres may not extend the entire length of these fascicles, and may instead be connected in series, it has been shown that fibres in this arrangement may activate simultaneously to act like single fibres [33]. It is therefore reasonable to assume that fascicles may function like single muscle fibres of equivalent length. These fibre bundles were mounted on glass slides and photographed under a microscope, and their lengths measured using ImageJ software, accurate to 0.01mm. This was repeated three times for each fibre of each muscle, and a mean value as well as standard deviations calculated. From these measurements, it was possible to calculate fibre length vs. muscle (belly) length ratio (L_f_:L_m_) for comparing architectural designs between muscles of different absolute lengths.

These variables were used to calculate PCSA (mm^2^) for each muscle using the following equation as described by Sacks and Roy [18]:

$$\text{PCSA }={\text{ (M}}_{\text{m}}\;*\text{ cos}\theta )/({\text{L}}_{\text{f}}\;*\;\rho ),$$

where M_m_ is muscle (belly) mass (g), L_f_ is muscle fibre length (mm), θ is muscle fibre pennation angle and ρ is the density of mammalian skeletal muscle (0.001056gmm^-3^) [34] (see [35] for a discussion on the validity of this value, which varies minimally in species measured to date). From these data, it was possible to calculate two architectural indices, PCSA:M_m_ and L_f_:PCSA, which have been used previously to compare muscles in terms of their designs for particular functions [36]. High PCSA:M_m_ values suggest a muscle is adapted for high force production, while a muscle with a high L_f_:PCSA value suggests a design for high shortening velocity or range of motion.

The methods employed here follow standard conventions established in other musculotendinous architecture studies of rodent limbs [11, 12, 14, 16].

#### Statistical analysis

To elucidate the extent of any architectural specialisations between muscle functional groups of the mouse hindlimb, a one-way analysis of variance (ANOVA) was performed. Post hoc pairwise comparisons (Tukey’s multiple comparisons) were subsequently carried out on variables which showed significant differences in the initial one-way ANOVA. The variables tested were: muscle mass (M_m_), muscle length (L_m_), optimal fibre length (L_f_), fibre length muscle length ratio (L_f_:L_m_), pennation angle and PCSA, as well as the architectural indices PCSA:M_m_ and L_f_:PCSA. The values of these variables were averaged between paired hindlimbs, giving a total of n = 4 for the statistical analysis. The ANOVA was carried out using statistics software SPSS (IBM, Armonk, NY), with the threshold of statistical significance defined as P<0.05.

## Results

### Musculoskeletal Geometry

Reconstructed microCT images captured are shown in Fig 1, where it can be seen that the contrast of the hindlimb soft tissue was sufficient to allow individual muscles to be discerned. Individual muscles were digitally segmented into discrete elements, which were used to construct a 3D representation of the mouse hindlimb (Fig 2). From the microCT images and the 3D model, a total of 39 muscles were observed and classed into nine functional groups (Tables 1 and 2). This grouping of muscles was based on a moment arm analysis carried out on a musculoskeletal model of the mouse hindlimb and pelvis [21], where a muscle’s most prominent function was assumed to be that which had the greatest moment arm value. Muscles were named based on those identified in previous studies of mouse hindlimb anatomy [10, 11], similar quadrupeds [14] or analogous muscles in the human leg [36–39].

**Table 1:** Attachment points of muscles within four proximal functional groups.

Muscle	Functional Group	Abbreviation	Origin	Insertion
**Gluteus maximus**	**Hip rotators**	**GM**	Lateral aspect of iliac crest	Lateral aspect of proximal femur
**Obturator externus**	**Hip rotators**	**OE**	Ventral aspect of caudal pubic ramus	Caudal aspect of greater trochanter
**Obturator internus**	**Hip rotators**	**OI**	Pubic tubercle	Medial aspect of greater trochanter
**Gemellus**	**Hip rotators**	**GEM**	Ischial spine	Medial aspect of greater trochanter (superior to obturator internus)
**Quadratus femoris**	**Hip rotators**	**QF**	Pubic tubercle (inferior to obturator internus)	Lateral aspect of mid-femur
**Tensor fascia latae**	**Hip rotators**	**TFL**	Cranial-medial aspect of iliac crest	Superior aspect of head of the fibula as the iliotibial tract
**Adductor magnus**	**Hip adductors**	**AM**	Cranial aspect of caudal pubic ramus	Caudal aspect of distal third of femur
**Adductor longus**	**Hip adductors**	**AL**	Cranial-medial aspect of cranial pubic ramus	Medial aspect of medial femoral condyle
**Adductor brevis**	**Hip adductors**	**AB**	Cranial aspect of cranial pubic ramus	Medial aspect of distal femur
**Gracilis (posterior)**	**Hip adductors**	**GP**	Body and caudal ramus of pubis	Medial aspect of proximal tibia
**Gracilis (anterior)**	**Hip adductors**	**GA**	Cranial aspect of body of pubis (caudal to adductor longus)	Medial aspect of proximal tibia
**Psoas major**	**Hip flexors**	**PMA**	Bodies of lower vertebrae	Lesser trochanter of the femur
**Psoas minor**	**Hip flexors**	**PMI**	Bodies of lower vertebrae, caudal to psoas major	Lesser trochanter of the femur
**Iliacus**	**Hip flexors**	**ILI**	Ventral aspect of iliac crest	Lesser trochanter of the femur
**Pectineus**	**Hip flexors**	**PECT**	Cranial aspect of the cranial pubic ramus	Medial aspect of the proximal femur
**Caudofemoralis**	**Hip extensors**	**CA**	Cranial facet of ischial tuberosity	Caudal-medial aspect of medial femoral condyle
**Semimembranosus**	**Hip extensors**	**SM**	Mid facet of ischial tuberosity	Medial aspect of proximal tibia (proximal to gracilis)
**Semitendinosus**	**Hip extensors**	**ST**	Caudal facet of ischial tuberosity	Medial aspect of proximal tibia (distal to gracilis)
**Biceps femoris (anterior)**	**Hip extensors**	**BFA**	Cranial facet of ischial tuberosity (superficial to caudofemoralis)	Caudal-medial aspect of lateral femoral condyle
**Biceps femoris (posterior)**	**Hip extensors**	**BFP**	Lower-mid facet of ischial tuberosity (superficial to semitendinosus)	Proximolateral aspect of the head of fibula and adjacent fascia

**Table 2:** Attachment points of muscles within two proximal and three distal functional groups.

Muscle	Functional Group	Abbreviation	Origin	Insertion
**Popliteus**	**Knee flexors**	**POP**	Caudal aspect of lateral femoral condyle	Caudal aspect of medial tibial condyle
**Rectus femoris**	**Knee extensors**	**RF**	Cranial inferior iliac spine of ilium	Base (proximal surface) of patella
**Vastus medialis**	**Knee extensors**	**VM**	Medial aspect of proximal part of femur	Base (proximal surface) of patella
**Vastus lateralis**	**Knee extensors**	**VL**	Lateral aspect of proximal part of femur	Base (proximal surface) of patella
**Vastus intermedius**	**Knee extensors**	**VI**	Cranial aspect of proximal part of femur	Base (proximal surface) of patella
**Patellar tendon**	**Knee extensors**	**PAT**	Apex (distal surface) of patella	Tibial tuberosity
**Tibialis anterior**	**Ankle dorsiflexors**	**TA**	Cranial-lateral aspect of proximal part of tibia	Medial cuneiform and base of 1st metatarsal
**Extensor digitorum longus**	**Ankle dorsiflexors**	**EDL**	Cranial aspect of proximal part of fibula	Dorsal surface of distal phalanges of digits 2–5
**Extensor hallucis longus**	**Ankle dorsiflexors**	**EHL**	Cranial-lateral aspect of mid part of tibia and adjacent membrane	Dorsal surface of distal phalanx of digit 1
**Medial gastrocnemius**	**Ankle plantarflexors**	**MG**	Medial supracondylar ridge of femur	Caudal surface of calcaneus via calcaneal tendon
**Lateral gastrocnemius**	**Ankle plantarflexors**	**LG**	Lateral supracondylar ridge of femur	Caudal surface of calcaneus via calcaneal tendon
**Soleus**	**Ankle plantarflexors**	**SOL**	Caudal aspect of upper- mid part of fibula	Caudal surface of calcaneus via calcaneal tendon
**Plantaris**	**Ankle plantarflexors**	**PLANT**	Lateral supracondylar ridge of femur (medial to lateral gastrocnemius)	Caudal surface of calcaneus via calcaneal tendon
**Flexor digitorum longus**	**Ankle plantarflexors**	**FDL**	Caudal aspect of medial tibial condyle	Plantar surface of distal phalanges digits 1–5
**Tibialis posterior**	**Ankle plantarflexors**	**TP**	Caudal aspect of proximal part of tibia	Tubercle of navicular
**Peroneus longus**	**Ankle everters**	**PL**	Lateral aspect of head of fibula	Plantar surface of medial cuneiform
**Peroneus brevis**	**Ankle everters**	**PB**	Caudal-lateral aspect of mid portion of fibula	Base of 5th metatarsal
**Peroneus tertius**	**Ankle everters**	**PT**	Caudal-lateral aspect of proximal portion of fibula	Base of cuboid
**Peroneus digiti quarti**	**Ankle everters**	**PDQA**	Caudal-lateral aspect of proximal portion of fibula (deep to peroneus tertius)	Dorso-lateral aspect of distal phalanx of digit 4
**Peroneus digiti quinti**	**Ankle everters**	**PDQI**	Caudal-lateral aspect of upper-mid-portion of fibula	Dorso-lateral aspect of distal phalanx of digit 5

Hip rotators consisted of M. gluteus maximus (GM), M. obturator externus (OE), M. obturator internus (OI), M. gemellus (GEM), M. quadratus femoris (QF) and M. tensor fascia latae (TFL). Previous studies of rodent hindlimb muscle architecture [14] have found multiple distinct muscles present within the gluteals group, however it was not possible here to accurately identify these. GM therefore refers to the entire group of gluteal muscles here. The TFL was continuous with the iliotibial tract, and was seen to contain very few muscle fibres. Therefore it was treated here as purely tendinous and only M_t_ and L_t_ were calculated. Hip adductors included M. adductor magnus (AM), M. adductor longus (AL), M. adductor brevis (AB) and M. gracilis (posterior, GP; and anterior, GA). Classed as hip flexors were M. psoas major (PMA), M. psoas minor (PMI), M. iliacus (ILI) and M. pectineus (PECT). Hip extensors included M. caudofemoralis (CF), M. semitendinosus (ST), M. semimembranosus (SM) and M. biceps femoris (anterior, BFA; posterior, BFP). The only muscle classed solely as a knee flexor was M. popliteus (POP). Knee extensors consisted of M. rectus femoris (RF), M. vastus medialis (VM), M.vastus lateralis (VL) and M. vastus intermedius (VI). Ankle dorsiflexors included M. tibialis anterior (TA), M. extensor digitorum longus (EDL) and M. extensor hallucis longus (EHL). Classed as ankle plantarflexors were M. gastrocnemius (medial, MG; lateral, LG), M. soleus (SOL), M. plantaris (PLANT), M. flexor digitorum longus (FDL) and M. tibialis posterior (TP). Finally, M. peroneus longus (PL), M. peroneus brevis (PB), M. peroneus tertius (PT), M. peroneus digiti quarti (PDQA) and M. peroneus digiti quinti (PDQI) were grouped together as ankle everters. It is recognised that many muscles are multi-articular (e.g. ST, SM, GA, GP etc.), so have prominent actions at two or more joints. In these cases, the muscles were grouped based on the most proximal joint on which they act. Exceptions to this included RF, which crossed the hip as well as the knee joint, and was classed as a knee extensor along with the other muscles of the quadriceps group (VM, VL and VI). The MG, LG and PLANT are also exceptions to this rule, as they were classed as ankle plantarflexors along with the other muscle of the ‘triceps surae’ group, SOL, despite crossing both the knee and ankle joints.

### Muscle architecture

The 39 muscles of the mouse hindlimb and pelvis identified from digital segmentation (see Tables 1 and 2) were dissected in order to determine their architectural properties. The average values of the variables measured from the eight hindlimbs of four mice are shown in Tables 3 and 4.

**Table 3:** Mean (±S.D) architectural properties of twenty hindlimb muscles, plus functional group means.

Muscle	M_m_ (mg)	M_m_+M_t_ (mg)	L_m_ (mm)	L_m_+L_t_ (mm)	L_f_ (mm)	L_f_:L_m_	Pennation angle (°)	PCSA (mm^2^)	PCSA:M_m_	L_f_:PCSA
**Gluteus maximus**	137.60±20.17	137.60±20.17	11.67±2.50	11.67±2.50	8.62±1.20	0.76±0.16	20.42±3.72	14.16±5.32	0.10±0.02	0.61±0.24
**Obturator externus**	0.75±0.35	0.75±0.35	3.83±0.39	3.83±0.39	2.85±0.79	0.81±0.09	0.00	0.25±0.12	0.33±0.50	11.44±6.07
**Obturator internus**	6.25±0.35	6.25±0.35	4.73±0.21	4.73±0.21	4.26±1.26	0.93±0.04	0.00	1.39±0.08	0.22±0.01	3.07±0.17
**Gemellus**	0.90±0.20	0.90±0.20	2.44±0.82	2.44±0.82	1.84±0.64	0.75±0.12	0.00	0.46±0.09	0.51±0.10	3.97±1.20
**Quadratus femoris**	33.25±1.48	33.25±1.48	10.75±1.13	10.75±1.13	8.93±2.96	0.90±0.10	0.00	3.52±0.16	0.11±0.01	2.53±0.11
**Tensor fascia latae**	N/A	83.5±3.60	N/A	15.82±1.53	N/A	N/A	N/A	N/A	N/A	N/A
**Hip rotators (mean)**	35.75±4.51	43.71±4.36	6.68±1.01	8.21±1.10	5.30±1.39	0.83±0.10	4.08±0.74	3.96±1.15	0.27±0.31	4.32±1.56
**Adductor magnus**	16.44±5.46	16.44±5.46	11.07±0.91	11.07±0.91	7.99±0.98	0.72±0.06	0.00	1.97±0.67	0.12±0.02	4.62±1.99
**Adductor longus**	10.55±4.28	10.55±4.28	9.84±1.88	9.84±1.88	7.40±1.03	0.77±0.11	0.00	1.32±0.41	0.13±0.02	6.09±2.01
**Adductor brevis**	5.30±1.53	5.30±1.53	8.13±1.98	8.13±1.98	6.49±1.45	0.80±0.06	0.00	0.81±0.27	0.15±0.03	9.51±5.79
**Gracilis (posterior)**	11.09±2.54	11.09±2.55	10.85±1.25	10.86±1.25	7.50±1.23	0.70±0.13	0.00	1.43±0.38	0.13±0.02	5.65±2.09
**Gracilis (anterior)**	12.50±3.04	12.50±3.04	12.60±1.18	12.60±1.18	7.65±1.87	0.61±0.13	0.00	1.59±0.36	0.13±0.03	5.11±1.91
**Hip adductors (mean)**	11.18±2.22	11.18±2.22	10.50±0.74	10.50±0.74	7.41±0.71	0.72±0.04	0.00	1.42±0.23	0.13±0.01	6.20±1.80
**Psoas major**	32.85±14.75	33.84±15.04	9.88±1.71	9.88±1.71	5.56±1.03	0.57±0.09	15.54±3.08	5.35±1.72	0.17±0.03	1.17±0.52
**Psoas minor**	22.04±6.78	22.04±6.87	9.90±1.38	9.90±1.38	5.63±1.30	0.58±0.17	12.57±7.11	3.95±2.13	0.17±0.05	1.84±1.09
**Iliacus**	16.56±7.15	16.56±7.15	9.59±1.51	9.59±1.51	6.99±0.69	0.75±0.09	0.00	2.29±1.06	0.14±0.01	4.64±4.83
**Pectineus**	3.54±1.09	3.54±1.09	5.98±1.27	5.98±1.27	3.58±0.84	0.61±0.15	15.18±2.10	0.93±0.25	0.27±0.08	4.23±1.95
**Hip flexors (mean)**	18.75±5.50	18.99±5.50	8.84+1.05	8.84±1.05	5.44±0.66	0.63±0.09	10.82±2.50	3.13±1.07	0.19±0.03	2.97±1.45
**Caudofemoralis**	22.16±4.98	22.15±4.98	14.13±0.72	14.13±0.72	11.15±1.44	0.79±0.10	0.00	1.90±0.43	0.09±0.01	6.18±1.63
**Semimembranosus**	78.61±9.92	78.61±9.92	15.72±0.55	15.72±0.55	10.92±0.54	0.69±0.04	0.00	6.81±0.76	0.09±0.00	1.62±0.19
**Semitendinosus**	50.79±7.21	50.79±7.21	17.74±1.38	17.74±1.38	11.00±1.61	0.63±0.09	0.00	4.34±0.78	0.09±0.01	2.61±0.63
**Biceps femoris (anterior)**	35.34±17.39	35.34±17.39	13.69±2.13	13.69±2.13	10.34±2.34	0.75±0.12	0.00	3.18±1.33	0.09±0.03	3.82±1.81
**Biceps femoris (posterior)**	88.45±19.62	88.45±19.62	16.68±0.85	16.68±0.85	11.11±1.33	0.67±0.06	0.00	7.90±1.56	0.09±0.01	1.44±0.26
**Hip extensors (mean)**	55.07±7.49	55.07±7.49	15.59±0.44	15.59±0.44	10.90±0.72	0.71±0.04	0.00	4.83±0.65	0.09±0.01	3.13±0.71

M_m_, muscle (belly) mass; M_t_, tendon mass; L_m_, muscle (belly) length; L_t_, muscle tendon length; L_f_, fibre length; L_f_:L_m_, fibre length vs. muscle length ratio; PCSA, physiological cross-sectional area; PCSA:M_m_, PCSA vs. muscle (belly) mass index; L_f_:PCSA, fibre length vs. PCSA index. Tensor fascia latae was continuous with the iliotibial tract and was mostly tendinous. Therefore no muscle properties were measured. For muscles with no tendons, M_m_ = M_m_+M_t_ and L_m_ = L_m_+L_t._

**Table 4:** Mean (±S.D) architectural properties of nineteen hindlimb muscles, plus functional group means.

Muscle	M_m_ (mg)	M_m_+M_t_ (mg)	L_m_ (mm)	L_m_+L_t_ (mm)	L_f_ (mm)	L_f_:L_m_	Pennation angle (°)	PCSA (mm^2^)	PCSA:M_m_	L_f_:PCSA
**Popliteus**	2.22±1.14	2.22±1.14	4.99±0.90	4.99±0.90	2.36±0.61	0.49±0.18	0.00	0.99±0.64	0.43±0.10	4.30±4.62
**Rectus femoris**	78.28±9.21	79.23±9.21	12.30±0.82	15.85±1.27	4.78±0.68	0.39±0.08	15.89±3.36	15.26±3.34	0.19±0.03	0.33±0.12
**Vastus medialis**	25.26±6.39	25.26±6.39	12.03±0.69	12.03±0.69	5.75+0.91	0.48±0.08	16.15±3.35	4.06±1.09	0.16±0.02	1.55±0.61
**Vastus lateralis**	67.80±14.19	67.79±14.20	13.06±1.05	13.06±1.05	6.51±0.89	0.50±0.07	15.63±4.25	9.58±2.16	0.14±0.02	0.71±0.20
**Vastus intermedius**	7.83±5.80	7.83±5.80	10.21±1.82	10.21±1.82	4.79±1.27	0.48±0.15	10.92±1.34	1.44±0.86	0.21±0.07	4.27±2.44
**Knee extensors (mean)**	44.79±6.34	45.03±6.34	11.90±0.69	12.79±0.63	5.46±0.39	0.46±0.04	14.65±2.95	7.59±1.36	0.18±0.02	1.72±0.62
**Tibialis anterior**	42.17±6.33	44.68±7.71	11.29±0.93	18.09±1.62	5.28±0.96	0.47±0.07	16.58±2.89	7.64±1.52	0.18±0.03	0.73±0.28
**Extensor digitorum longus**	8.23±1.93	8.96±2.11	11.08±2.21	25.67±2.37	5.04±0.48	0.48±0.13	12.39±2.12	1.52±0.38	0.18±0.02	3.53±0.99
**Extensor hallucis longus**	1.44±0.73	1.72±0.89	6.57±0.74	15.51±6.73	3.86±1.07	0.58±0.13	9.56±1.69	0.35±0.14	0.26±0.11	13.26±8.11
**Ankle dorsiflexors (mean)**	17.28±2.71	18.45±9.19	9.64±0.88	19.76±3.53	4.73±0.66	0.51±0.02	12.84±2.39	3.17±1.02	0.21±0.06	5.84±2.49
**Medial gastrocnemius**	33.89±4.22	34.15±4.50	10.46±0.51	15.44±0.79	4.25±0.60	0.41±0.06	14.24±2.68	7.43±1.41	0.22±0.03	0.60±0.17
**Lateral gastrocnemius**	72.0±11.84	72.7±11.85	11.76±0.54	16.09±0.88	4.49±0.47	0.38±0.04	17.28±2.73	14.57±2.54	0.20±0.02	0.32±0.08
**Soleus**	6.58±2.09	7.89±1.43	9.23±0.90	14.98±1.23	4.43±0.85	0.48±0.11	11.43±3.11	1.42±0.50	0.22±0.04	3.56±1.51
**Plantaris**	13.35±2.56	13.92±2.64	11.46±1.01	15.66±1.33	3.41±0.84	0.29±0.06	17.10±2.08	3.67±0.91	0.28±0.07	1.00±0.38
**Flexor digitorum longus**	28.78±3.66	34.91±4.39	13.15±0.62	28.28±2.33	3.74±0.91	0.28±0.07	15.20±2.50	7.30±1.46	0.26±0.06	0.55±0.22
**Tibialis posterior**	6.94±2.38	7.31±2.44	10.32±1.39	16.43±1.44	3.03±0.65	0.30±0.08	15.44±1.79	2.15±0.89	0.31±0.06	1.59±0.61
**Ankle plantarflexors (mean)**	26.93±3.64	28.48±4.39	11.06±0.49	17.81±1.10	3.89±0.62	0.36±0.06	15.11±2.10	6.09±1.05	0.25±0.04	1.27±2.22
**Peroneus longus**	8.62±2.51	9.05±2.52	9.71±0.73	17.65±0.64	3.68±0.70	0.38±0.08	14.90±2.85	2.21±0.74	0.26±0.04	1.92±0.95
**Peroneus brevis**	3.19±2.58	3.72±2.97	9.50±1.10	15.66±4.93	2.83±0.25	0.30±0.05	11.46±2.24	1.06±0.87	0.33±0.03	3.72±1.87
**Peroneus tertius**	5.46±1.76	6.01±1.92	10.86±1.24	17.58±1.03	4.06±1.05	0.37±0.07	12.46±3.63	1.28±0.43	0.24±0.05	3.58±1.97
**Peroneus digiti quarti**	1.55±0.54	1.91±0.56	8.39±1.90	20.80±3.35	2.70±0.55	0.35±0.13	12.42±3.73	0.54±0.16	0.35±0.09	5.50±2.02
**Peroneus digiti quinti**	1.30±0.17	1.67±0.17	7.21±0.88	22.90±2.98	3.38±0.68	0.49±0.12	9.44±1.76	0.33±0.10	0.27±0.08	11.16±4.69
**Ankle everters (mean)**	4.02±1.13	4.47±1.15	9.13±0.73	18.92±1.30	3.33±0.60	0.38±0.07	12.14±1.87	1.09±0.24	0.29±0.05	5.18±1.50

M_m_, muscle (belly) mass; M_t_, tendon mass; L_m_, muscle (belly) length; L_t_, muscle tendon length; L_f_, fibre length; L_f_:L_m_, fibre length vs. muscle length ratio; PCSA, physiological cross-sectional area; PCSA:M_m_, PCSA vs. muscle (belly) mass index; L_f_:PCSA, fibre length vs. PCSA index.

#### General features

The data showed that GM had the greatest M_m_ (and M_m_+M_t_) of the mouse hindlimb (137.60±20.17mg; mean ± SD), while FDL had the greatest M_t_ (6.13±0.83mg). ST had the longest L_m_ (17.74±1.38mm), PDQI had the longest L_t_ (15.69±3.22mm) and FDL had the longest L_m_+L_t_ (28.28±2.33mm). CF had the longest L_f_ (11.15±1.44mm), while OI had the greatest L_f_:L_m_ ratio (0.93±0.04). GM had the highest pennation angle (20.42±3.72°); however, the other hip rotators, as well as all the hip adductors, hip extensors and knee flexors had little or no noticeable fibre pennation. RF was calculated as having the greatest PCSA (15.26±3.34mm^2^), owing to its short fibres and large mass. In terms of the architectural indices calculated, it was found that GEM had the highest PCSA:M_m_ value (0.51±0.10), while EHL had the greatest L_l_:PCSA value (13.26±8.11).

**Fig 3:**
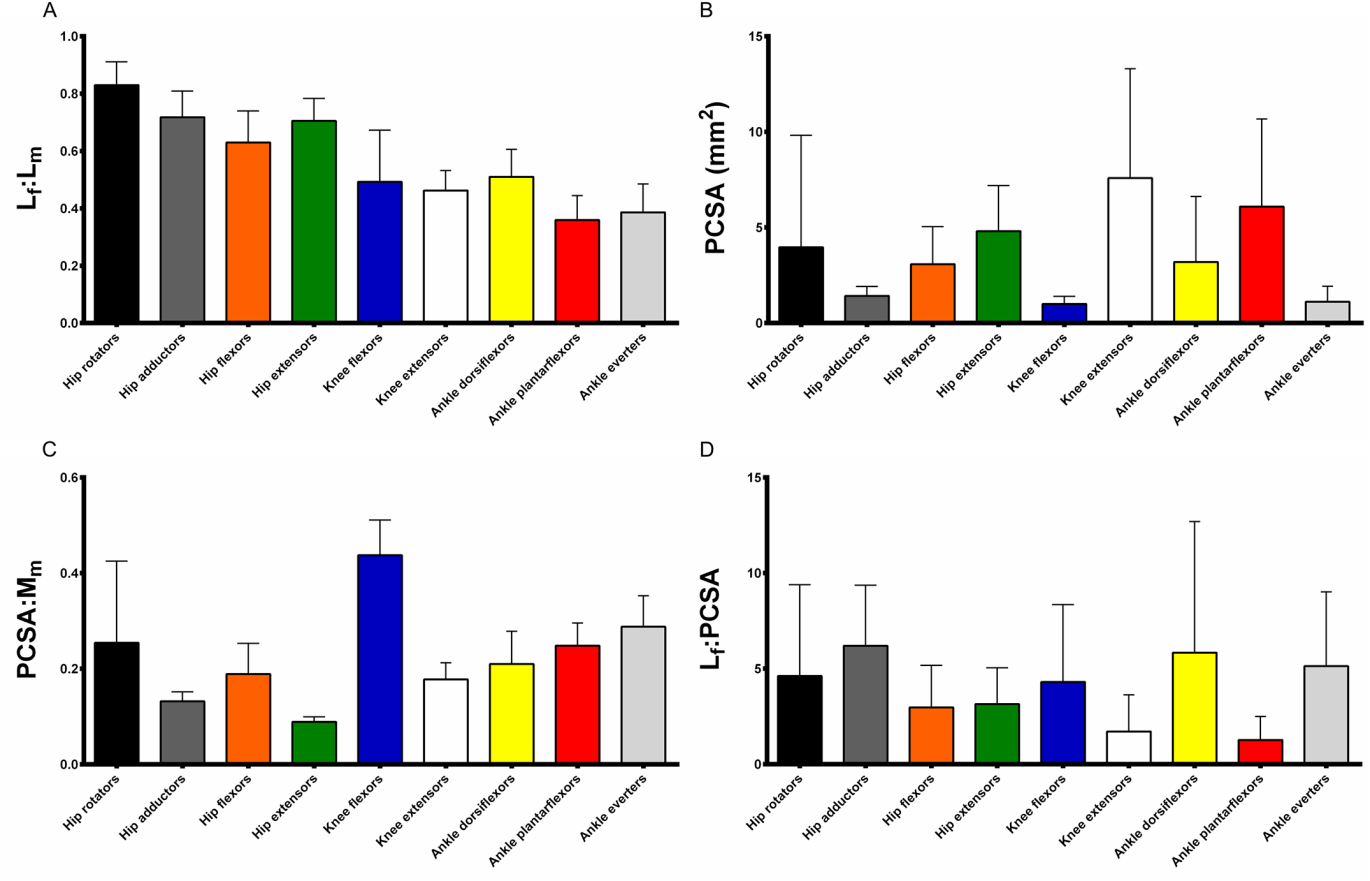
Functional group means of select muscle architectural properties. L_f_:L_m_, fibre length vs. muscle (belly) length ratio (A); PCSA, physiological cross-sectional area (B); PCSA:M_m,_ PCSA vs. muscle mass index (C); L_f_:PCSA, fibre length vs. PCSA index (D). Bars represent mean ± standard deviation.

When the mean value for each variable was calculated within the functional groups (Fig 3), it was found that the hip extensors had the greatest muscle belly mass (55.07±7.49mg), length (15.59±0.44mm) and absolute fibre length (10.90±0.72mm). The hip rotators on average had the greatest L_f_:L_m_ value (0.83±0.10). The ankle plantarflexors had the greatest pennation angle (15.11±2.10°), while the knee extensors had on average the greatest PCSA (7.59±1.36mm^2^). The knee flexors had the highest PCSA:M_m_ index (0.43±0.10), and the hip adductors had the greatest L_f_:PCSA index (6.20±1.80).

#### Statistical analyses

The extent of the functional specialisations of mouse hindlimb muscles was tested using a one-way ANOVA with post hoc pairwise comparisons (Tukey’s multiple comparisons), where 130 statistically significant differences (p<0.05) were found between functional groups for the tested variables. The results show that all of the tested variables show statistically significant differences between various functional groups (Tables 5, 6 and 7).

**Table 5:** Statistically significant differences between muscle functional groups in terms of architectural properties (Mm, Lm and Lf).

Variable	Functional group	Hip rotators	Hip adductors	Hip flexors	Hip extensors	Knee flexors	Knee extensors	Ankle dorsiflexors	Ankle plantarflexors	Ankle everters
	**Hip rotators**	n/a	ns	ns	ns	ns	ns	ns	ns	0.006
	**Hip adductors**	ns	n/a	ns	<0.001	ns	<0.001	ns	ns	ns
	**Hip flexors**	ns	ns	n/a	<0.001	ns	0.029	ns	ns	ns
	**Hip extensors**	ns	<0.001	<0.001	n/a	0.001	ns	<0.001	0.002	<0.001
**M**_**m**_	**Knee flexors**	ns	ns	ns	0.001	n/a	0.023	ns	ns	ns
	**Knee extensors**	ns	<0.001	0.029	ns	0.023	n/a	0.040	ns	<0.001
	**Ankle dorsiflexors**	ns	ns	ns	<0.001	ns	0.040	n/a	ns	ns
	**Ankle plantarflexors**	ns	ns	ns	0.002	ns	ns	ns	n/a	0.026
	**Ankle everters**	0.006	ns	ns	<0.001	ns	<0.001	ns	0.026	n/a
	**Hip rotators**	n/a	ns	ns	<0.001	ns	0.002	ns	0.027	ns
	**Hip adductors**	ns	n/a	ns	<0.001	<0.001	ns	ns	ns	ns
	**Hip flexors**	ns	ns	n/a	<0.001	0.021	0.001	ns	0.019	ns
	**Hip extensors**	<0.001	<0.001	<0.001	n/a	<0.001	<0.001	<0.001	<0.001	<0.001
**L**_**m**_	**Knee flexors**	ns	<0.001	0.021	<0.001	n/a	<0.001	0.003	<0.001	0.007
	**Knee extensors**	0.002	ns	0.001	<0.001	<0.001	n/a	ns	ns	0.002
	**Ankle dorsiflexors**	ns	ns	ns	<0.001	0.003	ns	n/a	ns	ns
	**Ankle plantarflexors**	0.027	ns	0.019	<0.001	<0.001	ns	ns	n/a	0.038
	**Ankle everters**	ns	ns	ns	<0.001	0.007	0.002	ns	0.038	n/a
	**Hip rotators**	n/a	<0.001	ns	<0.001	ns	ns	ns	ns	ns
	**Hip adductors**	<0.001	n/a	<0.001	<0.001	<0.001	<0.001	<0.001	<0.001	<0.001
	**Hip flexors**	ns	<0.001	n/a	<0.001	<0.001	ns	ns	0.001	<0.001
	**Hip extensors**	<0.001	<0.001	<0.001	n/a	<0.001	<0.001	<0.001	<0.001	<0.001
**L**_**f**_	**Knee flexors**	ns	<0.001	<0.001	<0.001	n/a	<0.001	0.021	ns	ns
	**Knee extensors**	ns	<0.001	ns	<0.001	<0.001	n/a	ns	0.002	<0.001
	**Ankle dorsiflexors**	ns	<0.001	ns	<0.001	0.021	ns	n/a	ns	0.046
	**Ankle plantarflexors**	ns	<0.001	0.001	<0.001	ns	0.002	ns	n/a	ns
	**Ankle everters**	ns	<0.001	<0.001	<0.001	ns	<0.001	0.046	ns	n/a

P values calculated using one-way ANOVA and Tukey’s multiple comparisons. Statistical significance = p<0.05. M_m_, muscle (belly) mass; L_m_, muscle (belly) length; L_f_, fibre length; ns, not significant.

**Table 6:** Statistically significant differences between muscle functional groups in terms of architectural properties (Lf:Lm, pennation angle and PCSA).

Variable	Functional group	Hip rotators	Hip adductors	Hip flexors	Hip extensors	Knee flexors	Knee extensors	Ankle dorsiflexors	Ankle plantarflexors	Ankle everters
	**Hip rotators**	n/a	ns	ns	ns	ns	0.002	0.047	<0.001	<0.001
	**Hip adductors**	ns	n/a	ns	ns	0.013	<0.001	<0.001	<0.001	<0.001
	**Hip flexors**	ns	ns	n/a	ns	ns	0.002	ns	<0.001	<0.001
	**Hip extensors**	ns	ns	ns	n/a	0.025	<0.001	<0.001	<0.001	<0.001
**L**_**f**_**:L**_**m**_	**Knee flexors**	ns	0.013	ns	0.025	n/a	ns	ns	ns	ns
	**Knee extensors**	0.002	<0.001	0.002	<0.001	ns	n/a	ns	ns	ns
	**Ankle dorsiflexors**	0.047	<0.001	ns	<0.001	ns	ns	n/a	0.008	ns
	**Ankle plantarflexors**	<0.001	<0.001	<0.001	<0.001	ns	ns	0.008	n/a	ns
	**Ankle everters**	<0.001	<0.001	<0.001	<0.001	ns	ns	ns	ns	n/a
	**Hip rotators**	n/a	ns	0.041	ns	ns	<0.001	<0.001	<0.001	<0.001
	**Hip adductors**	ns	n /a	<0.001	ns	ns	<0.001	<0.001	<0.001	<0.001
	**Hip flexors**	0.041	<0.001	n/a	<0.001	0.001	ns	ns	0.001	ns
	**Hip extensors**	ns	ns	<0.001	n/a	ns	<0.001	<0.001	<0.001	<0.001
**Pennation angle**	**Knee flexors**	ns	ns	0.001	ns	n/a	<0.001	<0.001	<0.001	<0.001
	**Knee extensors**	<0.001	<0.001	ns	<0.001	<0.001	n/a	ns	ns	ns
	**Ankle dorsiflexors**	<0.001	<0.001	ns	<0.001	<0.001	ns	n/a	ns	ns
	**Ankle plantarflexors**	<0.001	<0.001	0.001	<0.001	<0.001	ns	ns	n/a	ns
	**Ankle everters**	<0.001	<0.001	ns	<0.001	<0.001	ns	ns	ns	n/a
	**Hip rotators**	n/a	ns	ns	ns	ns	ns	ns	ns	ns
	**Hip adductors**	ns	n/a	ns	0.043	ns	<0.001	ns	<0.001	ns
	**Hip flexors**	ns	ns	n/a	ns	ns	0.006	ns	ns	ns
	**Hip extensors**	ns	0.043	ns	n/a	ns	ns	ns	ns	0.018
**PCSA**	**Knee flexors**	ns	ns	ns	ns	n/a	0.016	ns	ns	ns
	**Knee extensors**	ns	<0.001	0.006	ns	0.016	n/a	0.021	ns	<0.001
	**Ankle dorsiflexors**	ns	ns	ns	ns	ns	0.021	n/a	ns	ns
	**Ankle plantarflexors**	ns	<0.001	ns	ns	ns	ns	ns	n/a	<0.001
	**Ankle everters**	ns	ns	ns	0.018	ns	<0.001	ns	<0.001	n/a

P values calculated using one-way ANOVA and Tukey’s multiple comparisons. Statistical significance = p<0.05. L_f_:L_m_, fibre length vs. muscle (belly) length index; PCSA, physiological cross-sectional area; ns, not significant.

**Table 7:** Statistically significant differences between muscle functional groups in terms of architectural properties (PCSA:M_m_ and L_f_:PCSA).

Variable	Functional group	Hip rotators	Hip adductors	Hip flexors	Hip extensors	Knee flexors	Knee extensors	Ankle dorsiflexors	Ankle plantarflexors	Ankle everters
	**Hip rotators**	n/a	ns	ns	0.001	<0.001	ns	ns	ns	ns
	**Hip adductors**	ns	n/a	ns	ns	<0.001	ns	0.014	<0.001	<0.001
	**Hip flexors**	ns	ns	n/a	<0.001	<0.001	ns	ns	ns	<0.001
	**Hip extensors**	0.001	ns	<0.001	n/a	<0.001	0.001	<0.001	<0.001	<0.001
**PCSA:M**_**m**_	**Knee flexors**	<0.001	<0.001	<0.001	<0.001	n/a	<0.001	<0.001	<0.001	<0.001
	**Knee extensors**	ns	ns	ns	0.001	<0.001	n/a	ns	0.010	<0.001
	**Ankle dorsiflexors**	ns	0.014	ns	<0.001	<0.001	ns	n/a	ns	0.014
	**Ankle plantarflexors**	ns	<0.001	ns	<0.001	<0.001	0.010	ns	n/a	ns
	**Ankle everters**	ns	<0.001	<0.001	<0.001	<0.001	<0.001	0.014	ns	n/a
	**Hip rotators**	n/a	ns	ns	ns	ns	ns	ns	ns	ns
	**Hip adductors**	ns	n/a	ns	ns	ns	0.002	ns	<0.001	ns
	**Hip flexors**	ns	ns	n/a	ns	ns	ns	ns	ns	ns
	**Hip extensors**	ns	ns	ns	n/a	ns	ns	ns	ns	ns
**L**_**f**_**:PCSA**	**Knee flexors**	ns	ns	ns	ns	n/a	ns	ns	ns	ns
	**Knee extensors**	ns	0.002	ns	ns	ns	n/a	0.034	ns	ns
	**Ankle dorsiflexors**	ns	ns	ns	ns	ns	0.034	n/a	0.004	ns
	**Ankle plantarflexors**	ns	<0.001	ns	ns	ns	ns	0.004	n/a	0.005
	**Ankle everters**	ns	ns	ns	ns	ns	ns	ns	0.005	n/a

P values calculated using one-way ANOVA and Tukey’s multiple comparisons. Statistical significance = p<0.05. PCSA:M_m_, physiological cross-sectional area vs. muscle (belly) mass index; L_f:_PCSA, fibre length vs. physiological cross-sectional area index; ns, not significant.

In terms of M_m_, the hip and knee extensors were significantly more massive than the majority of other functional groups. In addition, the hip rotators and ankle plantarflexors had significantly greater mass than the ankle everters. The hip and knee extensors were both significantly longer in terms of L_m_ than most other functional groups, while the knee flexors were significantly shorter. The ankle plantarflexors were significantly longer than the ankle everters. Most significant differences were found in terms of L_f_, where the hip adductors and extensors were significantly longer than all other groups. The hip flexors as well as the knee flexors and extensors were significantly longer than the more distal groups. Ankle dorsiflexors were also significantly longer than the ankle everters. The L_f_:L_m_ ratios of the hip functional groups were significantly longer than those of the muscles acting around the knee and ankle joints. The ankle dorsiflexors were significantly greater in L_f_:L_m_ than the plantarflexors. In terms of pennation angle, the proximal hip muscles were significantly different to the more distal knee and ankle muscles, owing to the more parallel fibre structure of the proximal muscles. The PCSA of the knee extensors was significantly higher than most of the other functional groups. The ankle plantarflexors’ PCSA values were also significantly greater than the ankle everters’. Significant differences were also found between functional groups for the two PCSA-related indices calculated. The PCSA:M_m_ of the knee flexors was found to be significantly greater than all the other groups. The hip extensors were significantly lower than most other groups, especially the distal ankle muscles (which is also the case with the hip adductors). The hip flexors and knee extensors were significantly lower than the distal groups in terms of PCSA:M_m_, while the ankle dorsiflexors were significantly lower than the ankle everters. The highly pennate knee extensors and ankle plantarflexors had significantly lower L_f_:PCSA than the hip adductors and ankle dorsiflexors. The ankle plantarflexors’ L_f_:PCSA values were also significantly lower than the ankle everters’.

## Discussion

The aim of this paper was to characterise the musculoskeletal geometry and architecture of hindlimb and pelvis of mice, and determine the extent to which the functional groups of muscles are architecturally specialised for their respective functions.

### Musculoskeletal geometry

Contrast-enhanced microCT scanning was used here as a less destructive method of determining the musculoskeletal geometry of the mouse hindlimb compared to dissections, due mostly to the small size and fragility of the muscles under investigation. It was found that soaking a detached mouse hindlimb specimen in an aqueous I_2_KI solution prior to microCT scanning produced remarkable soft tissue contrast, allowing individual muscles and in some cases fibre pennation angles to be observed, without the need to remove or damage other important structures. This replicates the findings of prior studies [23–31] which used similar techniques to investigate soft tissue anatomy. A total of 39 muscles of the hindlimb and pelvis were identified. Using the reconstructed microCT images, digital segmentation was carried out to create 3D meshes of each bone and musculotendon unit, allowing attachment sites and paths of action for all these muscles to be observed in an interactive environment. A fully interactive model can be viewed as a 3D PDF (S1 Fig). Simple guidance on manipulating this 3D model is given in the S1 Appendix.

### Musculotendon architecture

Six architectural variables (i.e. the properties of muscles which determine force-generating and length-change potential) of the 39 muscles of the mouse hindlimb and pelvis previously identified were determined through microdissections under high magnification, and physiological cross-section areas (PCSA) were calculated. The data and the results of the one-way ANOVA indicate that the functional groups exhibited very strong functional specialisation in terms of their architecture, which would allow each group to contribute differently to motor output during both locomotor and non-locomotor movements.

#### General features

The functional group averages show that the hip extensors had the greatest M_m_, L_m_ and L_f_, and lowest pennation angle, of all of the functional groups. They had on average significantly greater L_f_, L_f_:L_m_ and lower pennation angles than the distal muscles of the hindlimb, as well as the knee extensors. All of the other proximal muscle groups which act upon the hip (rotators, adductors and flexors) also displayed significantly higher L_f_:L_m_ values than the more distal groups. Hip adductors also showed the greatest L_f_:PCSA index [36], which was significantly greater than the more powerful groups such as the knee flexors and ankle plantarflexors. It is thought that architectural adaptations such as these, shown strongly here in the hip extensors and hip adductors, adapt a muscle for high contraction velocities [13] or range of motion. Given that contraction speed is a function of the number of in series sarcomeres within a fibre, functionally this allows a muscle with long fibres to undergo the maximum possible excursion during contraction with the lowest possible loss of force, and also permits the muscles to produce higher forces over a wider range of muscle lengths and shortening velocities than a similarly sized muscle with shorter fibres [13, 33].

The knee extensors, a group containing the quadriceps femoris muscles, on average had the significantly greatest PCSA of any functional group, suggesting these are the muscles adapted to produce the greatest absolute force. This capacity for high force-generation is also shared by the hip extensors and ankle plantarflexors. The presumed function of these three muscle groups in mouse locomotion is to overcome gravity and inertia during the swing phase, and provide support and stability to the limb during the stance phase. The fibre architectural design found in these muscles may allow them to provide high absolute forces around the respective joints and limb segments in order to carry out these functions [13].

PCSA:M_m_ index, which is an indicator of a muscle’s relative ability to produce force [36], was significantly greatest in the knee flexors, a group which here contained only M. popliteus (POP), relative to the majority of other functional groups. This adaptation for high relative force production is expected, as POP likely functions not only to assist in knee flexion but also stabilise the knee joint, where it may contract eccentrically to maintain the flexed hip and knee postures of the mouse during locomotion [40]. This was confirmed using a musculoskeletal model of a mouse hindlimb and pelvis [17], where POP was seen to have a ‘zero-crossing’ moment arm with a negative slope around the knee joint, which is thought to reveal that the muscle provides an intrinsic stabilisation to the joint [41].

#### Muscle classifications

As mentioned above, POP was the only muscle classed primarily as a flexor of the knee, although it was recognised that there are several bi-articular muscles which carry out functions at the knee joint, but were classified into other groups. Moment arm analysis of the mouse hindlimb muscles using a musculoskeletal model [17] confirmed that the bi-articular muscles of the ‘triceps surae’ group; M. gastrocnemius (medial and lateral) and M. plantaris, also assist in knee flexion. Similarly, the bi-articular hip adductors M. gracilis anterior and M. gracilis posterior, as well as the bi-articular hip extensors M. semimembranosus, M. semitendinosus and M. biceps femoris posterior, all produce large flexion moment arms at the knee, and could therefore also be classed as knee flexors. However these muscles were not classed as knee flexors, as they provide larger moment arms at other joints (the hip and ankle) [17]. Furthermore, bi-articular muscles are thought to carry out unique functions during movements relative to closely associated mono-articular muscles [42–44], so may possess unique architectural characteristics. Some justification may therefore exist for placing these muscles in their own functional group for future statistical analyses such as the one performed here. Ultimately, however, although the muscles of the mouse hindlimb and pelvis could be classified differently than done so here, given the number of redundant degrees of the freedom and the inherent over-actuation of the vertebrate musculoskeletal system in general, issues regarding muscle functional classifications are likely to arise in most instances when investigating anatomical specialisations between muscle functional groups, but in reality cannot be avoided.

#### Proximo-distal gradient of architecture

Of the 130 statistically significant differences that were found between the functional groups, 68 (52.30%) of these were between the proximal functional groups (hip rotators, hip adductors, hip extensors, knee extensors and knee flexors) and the distal groups (ankle dorsiflexors, plantarflexors and everters). The data therefore firmly indicate that a proximo-distal gradient of musculotendon architecture exists within the mouse hindlimb and pelvis, a phenomenon also observed in the hindlimbs of cursorial quadrupeds such as horses [45] racing greyhounds [46] and hares [47], non-cursorial tetrapods such as crocodiles [48, 49] as well as flightless birds [50, 51].

In the hindlimb of mice, the proximal muscle groups such as the hip extensors, hip flexors and knee extensors tended to exhibit greater M_m_, L_f_, L_f_:L_m_ and PCSA than more distal groups such as the ankle dorsiflexors and plantarflexors, which had greater pennation angles and PCSA:M_m_ values. These are similar to other reported findings of tetrapod hindlimb anatomy, although the distal reduction in mass and PCSA appear less pronounced in mice, something that was also reported in the hare [47]. This is most likely due to the more flexed hindlimb postures of these animals, meaning that an increase in power from the ankle plantarflexors may be needed during locomotion relative to more straight–limbed, cursorial quadrupeds, such horses [45, 46].

The functional significance of this ‘limb tapering’ has been heavily studied, and is thought to represent an adaptation for energetically efficient and stable locomotion over various terrains [22, 45, 50, 52–58]. Proximal limb muscles, such as the hip extensors with their long, parallel fibres, function to provide mechanical power, fast contractions and precise control of joint positions to overcome gravity and move the body’s centre of mass during locomotion. Muscles with smaller mass, shorter fibres, higher pennation angles and long compliant tendons are generally located distally within terrestrial limbs. Tendons act as mechanical buffers, absorbing energy from initial ground contact and subsequently using it to reduce the amount of work the muscle needs to perform to move limb segments through elastic recoil [22, 52, 58, 59]. Furthermore, a reduction in mass distally within the limb is useful as it minimises the moment of inertia acting on the limb during the swing phase of terrestrial locomotion [60].

These differences in intrinsic properties of hindlimb muscles are also thought to play a critical role in maintaining stability in response to sudden perturbations during fast locomotion, creating a similar proximo-distal gradient in neuromuscular control within the hindlimb and simplifying locomotor control. It is predicted that proximal muscles, with parallel fibres and non-tendinous attachments, will be relatively insensitive to unexpected perturbations, such as a change in terrain height, during locomotion. More distal muscles on the other hand, with substantial in-series tendon elasticity, have been discovered to show relatively high sensitivity to these perturbations, either increasing or decreasing their effects depending on the stage in the stride cycle in which perturbations occur [22, 58].

Although a proximo-distal gradient of musculoskeletal architecture in hindlimb was initially thought to characterise large, cursorial tetrapods, its appearance in small, non-cursorial animals such as the mouse is interesting. Mice are prey animals, and frequently utilise short bursts of high speed running to escape potential predators. These movements presumably require high work output from the proximal muscles to move the body quickly, and high power from the anti-gravity muscles groups to move the limb against gravity and inertia. Thus evolutionary pressure to optimise the limb for rapid movement is expected, but this pressure could be mitigated by an enhanced force/mass ratio that may come with smaller size. Evidence of a similar proximo-distal gradient in mice suggests that these evolutionary pressures for fast locomotion outweigh any gains in muscle force development capability relative to body/limb mass that may have come with the small body size (and under the assumption of isometric scaling) [61–64].

## Conclusions

This paper described in detail the musculoskeletal geometry and architecture of the mouse hindlimb and pelvis, and placed the architectural specialisations which exist between the functional groups of muscles into a functional context. Strong architectural specialisations exist between the functional groups, especially for variables which adapt a muscle for high contraction velocity, joint control and high mechanical work, which reflects their presumed functions during locomotion. A detailed moment arm analysis of mouse hindlimb and pelvis musculature, based on a concurrently developed mouse hindlimb musculoskeletal model [17], supports these assumed functions, and further establishes the functional significance of the muscle architectural characteristics described here.

Our data confirm the presence of a proximo-distal gradient of architectural adaptation within the mouse hindlimb, moving from muscles with long fibres and low pennation angles to muscles showing highly pennate fibre arrangements and compliant, tendinous insertions as the limb is traversed proximo-distally. This is thought to be an adaptation to improve locomotor efficiency, motor coordination, and enhance resistance to external perturbations, and is commonly associated with larger, more cursorial terrestrial vertebrates [53, 58]. The appearance in the small, non-cursorial mouse suggests that this adaptation for energy saving and resistance to perturbations during locomotion extends far beyond larger, cursorial or bipedal organisms and may, to a degree, characterize most extant tetrapods (e.g. [48–50]).

## Supporting Information

S1 Appendix Quick-start guide to using the 3D pdf of the mouse hindlimb and pelvis. (PDF)

S1 Fig Three Dimensional pdf of the mouse hindlimb and pelvis. (PDF)
