# Supplementary material for: Musculoskeletal Geometry, Muscle Architecture and Functional Specialisations of the Mouse Hindlimb
Source: PLoS One. 2016 Apr 26;11(4):e0147669. doi: 10.1371/journal.pone.0147669 (PMC4846001; doi:10.1371/journal.pone.0147669)

Quick-start guide to using the 3D pdf of the mouse hindlimb and pelvis

Once open, a 3D representation of mouse hindlimb and pelvis should appear in a cranial-medial view. To **zoom in/out**, either **scroll with the middle mouse**, or **right click and hold** and move the mouse up/down. To **rotate** the model, **left click and hold** and move the mouse in the desired direction.

To **add/remove** bones or muscles to simulate a digital dissection, the **model tree** needs to be open. If not open, go to **View->Show/hide->Navigation panes** and select **Model Tree** (pictured). Within the model tree, extend the **model and root** drop down menus, revealing a list of mouse hindlimb structures, which are all turned on by default.

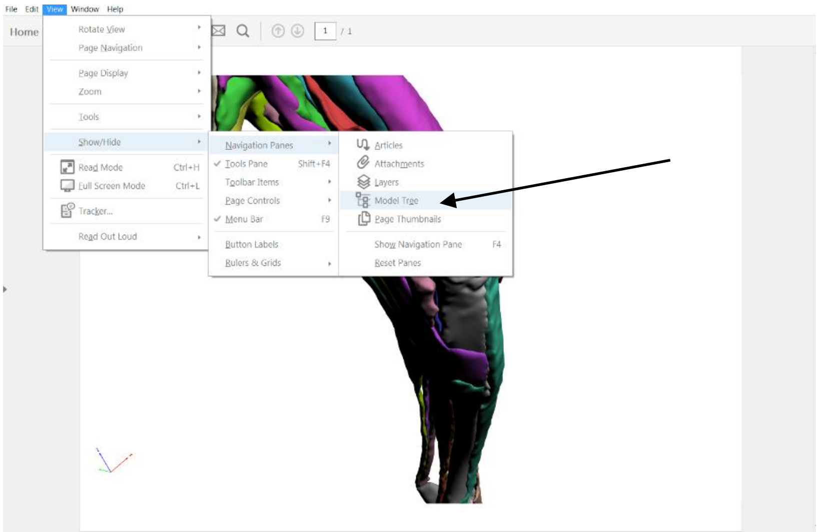

Uncheck/check the boxes next to each structure to turn it off/on and observe the deeper structures of the mouse hindlimb.

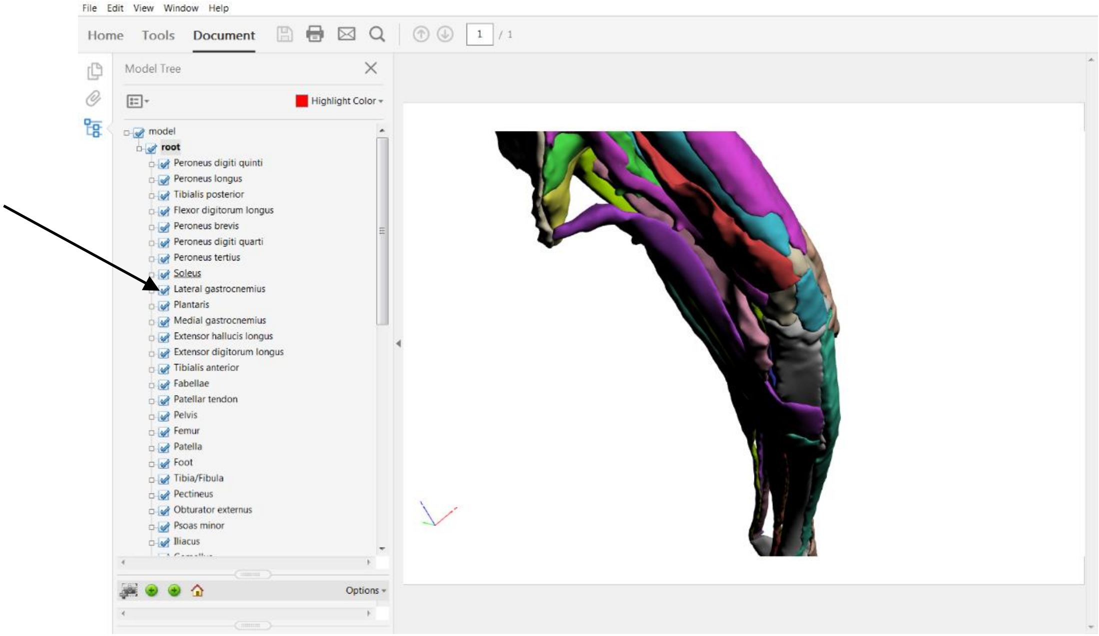

Supplement: S1 Appendix — (PDF) [file pone.0147669.s001.pdf]
